# Bioinformatics-based and multiscale convolutional neural network screening of herbal medicines for improving the prognosis of liver cancer: a novel approach

**DOI:** 10.3389/fmed.2023.1218496

**Published:** 2023-08-23

**Authors:** Zeshan Chen, Peichun Peng, Miaodong Wang, Xin Deng, Rudi Chen

**Affiliations:** ^1^Department of Traditional Chinese Medicine, People's Hospital of Guangxi Zhuang Autonomous Region, Nanning, China; ^2^International Zhuang Medicine Hospital, Guangxi University of Traditional Chinese Medicine, Nanning, China; ^3^Guangxi University of Traditional Chinese Medicine, Nanning, China; ^4^Basic Medical College of Guangxi University of Traditional Chinese Medicine, Nanning, China

**Keywords:** hepatocellular carcinoma, deep learning, convolutional neural network, traditional Chinese medicine, gene, genetic algorithm, bioinformatics

## Abstract

**Background:**

Liver cancer is one of the major diseases threatening human life and health, and this study aims to explore new methods for treating liver cancer.

**Methods:**

A deep learning model for the efficacy of clinical herbal medicines for liver cancer was constructed based on NDCNN, combined with the natural evolutionary rules of a genetic algorithm to obtain the herbal compound for liver cancer treatment. We obtained differential genes between liver cancer tissues and normal tissues from the analysis of TCGA database, screened the active ingredients and corresponding targets of the herbal compound using the TCMSP database, mapped the intersection to obtain the potential targets of the herbal compound for liver cancer treatment in the Venny platform, constructed a PPI network, and conducted GO analysis and KEGG analysis on the targets of the herbal compound for liver cancer treatment. Finally, the key active ingredients and important targets were molecularly docked.

**Results:**

The accuracy of the NDCNN training set was 0.92, and the accuracy of the test set was 0.84. After combining with the genetic algorithm for 1,000 iterations, a set of Chinese herbal compound prescriptions was finally the output. A total of 86 targets of the herbal compound for liver cancer were obtained, mainly five core targets of IL-6, ESR1, JUN, IL1β, and MMP9. Among them, quercetin, kaempferol, and stigmasterol may be the key active ingredients in hepatocellular carcinoma, and the herbal compound may be participating in an inflammatory response and the immune regulation process by mediating the IL-17 signaling pathway, the TNF signaling pathway, and so on. The anticancer effects of the herbal compound may be mediated by the IL-17 signaling pathway, the TNF signaling pathway, and other signaling pathways involved in inflammatory response and immune regulation. Molecular docking showed that the three core target proteins produced stable binding to the two main active ingredients.

**Conclusion:**

The screening of effective herbal compounds for the clinical treatment of liver cancer based on NDCNN and genetic algorithms is a feasible approach and will provide ideas for the development of herbal medicines for the treatment of liver cancer and other cancers.

## 1. Introduction

Primary liver cancer, including three types of hepatocellular carcinoma, cholangiocarcinoma, and mixed carcinoma, was the fourth leading cause of cancer death in 2018. Its prognosis is poor, with a 5-year survival rate of ≤ 5% for advanced liver cancer, which is a serious threat to human life and health (1). The main causes of liver cancer include metabolic disorders, chronic infection with the hepatitis virus, excessive alcohol consumption, and excessive aflatoxin intake (2). According to statistics (3), East Asia has the highest number of liver cancer patients, with Australia having the fastest-growing incidence of liver cancer. China has a high prevalence of hepatitis B, resulting in a significant number of liver cancer patients associated with the virus (4). Currently, there are several drug treatments for liver cancer, such as targeted drug therapy, immunotherapy, and chemotherapy, but these methods have limitations, such as incomplete treatment, easy recurrence after treatment, and bone marrow suppression. Therefore, it is of great significance to find other effective, safe, and precise treatment methods.

With its long-standing history in China, Chinese medicine has been widely considered due to its potent anticancer properties and minimal adverse effects associated with the active ingredients found in traditional Chinese medicine. The monomer or other effective active substances extracted from traditional Chinese medicine have multi-target and low-toxicity anti-tumor effects (5). However, how to screen multi-target, multi-pathway, and low-toxicity anti-tumor drugs is a major challenge at present (6).

Screening key hepatocellular carcinoma genes as core targets will help improve the therapeutic efficacy of hepatocellular carcinoma (7). The development of bioinformatics technologies has driven comprehensive cancer gene research (8, 9). The large amount of data generated by new technologies such as genome sequencing and microarrays makes data management and multi-platform integration important, which will provide a research platform for researchers worldwide. Jiang et al. (10) found that CDNK3 was the key gene in the progression of cirrhosis to hepatocellular carcinoma. In addition, Urh et al. (11) bioinformatically analyzed normal mucosal, colorectal adenoma, and colorectal cancer differential genes from GEO as well as The Cancer Genome Atlas (TCGA) databases and found that tumor stem cell-related genes (ANLN, CDK1, ECT2, and TNC) were associated with colorectal carcinogenesis, while ANLN and PDGFD genes were associated with the progression of colorectal cancer, but the mechanism of action of these genes with the development of colorectal cancer still needs further study and validation.

Artificial intelligence (AI) is a field that encompasses theoretical, methodological, and applied techniques to simulate, extend, and expand human intelligence. It finds application in various domains, including medical care, where AI is actively applied (12). The establishment of drug prediction models based on clinical big data is the current research innovation in the intelligence of Chinese medicine-assisted diagnosis and treatment (13). The use of computer-aided diagnosis and treatment technology for herbal medicine recommendation is a textual multi-label classification problem (14), and the commonly used methods are machine learning (ML) and deep learning (DL). A convolutional neural network (CNN) belongs to a branch of DL and is a common text classification model consisting of several parts: an input layer, a convolutional layer, a pooling layer, a fully connected layer, and an output layer, the convolutional layer of which with local linking, weight sharing, and pooling operations can both effectively extract features and reduce parameters in the network, thus substantially simplifying the complexity of the network (15). Therefore, we used the CNN models to fit the intrinsic link between clinical efficacy and prescription to improve drug prediction accuracy.

Cyberpharmacology integrates biology, pharmacology, mathematics, and computer science. It generally integrates biological networks and drug action networks with the help of biological network databases and drug databases; establishing a “drug-disease-target-gene” interaction network can allow one to observe the drug action on the disease target at the overall level. This study is based on a multi-scale convolutional neural network.

In this study, we screened Chinese herbal medicines based on a multi-scale convolutional neural network model (N-Dim CNN and NDCNN) and genetic algorithm to improve the prognosis of liver cancer, used the TACG database to screen poor prognosis genes as candidate target genes, and conducted network pharmacology and molecular docking between the screened drugs and the candidate target genes to demonstrate that the screened drugs may improve the prognosis of liver cancer patients by targeting poor prognosis genes. The prognosis of hepatocellular carcinoma patients provides a new approach for the development of herbal medicines for the treatment of hepatocellular carcinoma and other cancers.

## 2. Screening of Chinese medicines

### 2.1. Data sources

In this study, we obtained a total of 745 pieces of literature and clinical cases using TCM for primary liver cancer from four major databases, namely, the Chinese Journal Full Text Database, the Chinese Science and Technology Journal Full Text Database, the Wanfang Data Knowledge Platform, and the Rui Kang Hospital affiliated to Guangxi University of Traditional Chinese Medicine, and extracted these 745 TCM treatment protocols and clinical efficacy data. There were four types of outcomes after treatment: complete remission: the tumor had completely disappeared; partial remission: the tumor size reduced by more than 50%; stable: the tumor size reduced by <50% or increased by <25%; progressive: the tumor size increased by more than 25% or new lesions appeared. Among them, there were nine articles or cases with a treatment effect of complete remission, 95 partial remissions, 513 stable cases, and 128 progressive cases. The study was approved by the Medical Ethics Committee of Ruikang Hospital, Guangxi University of Traditional Chinese Medicine, Nanning, Guangxi, China.

### 2.2. Data preprocessing

The 745 cases of herbal treatment protocols and treatment effects were extracted to an Excel sheet, and the herbal terminology was standardized according to the standard of “Traditional Chinese Medicine” (Chinese Traditional Medicine Press, 2007 edition). Each different Chinese medicine was recorded and represented as a single entry within an Excel cell after normalization. Finally, 334 different TCMs were obtained for the treatment of primary liver cancer after normalization. Using the bag-of-words vector model coding, each herbal medicine appearing in the prescription was then identified with 1 binary bit, and the presence of that herbal medicine was marked as 1, and its absence was noted as 0 (see Table 1).

**Table 1:** Data codes.

**Number**	***Astragalus***	**Thorowax Root**	***Rhizoma Corydalis***	**Nutgrass Galingale Rhizome**	***Mylabris phalerata* Pallas**	**…**	***Hedyotis diffusa* Willd**	**Rhizoma Paridis**
	**S1**	**S2**	**S3**	**S4**	**S5**	**…**	**S334**	**S334**
1	0	1	0	1	1	**…**	0	0
2	0	1	1	1	0	**…**	0	0
3	0	1	1	0	0	**…**	0	1
**…**	**…**	**…**	**…**	**…**	**…**	**…**	**…**	**…**
**…**	**…**	**…**	**…**	**…**	**…**	**…**	**…**	**…**
744	1	0	0	1	0	**…**	0	0
745	0	0	1	1	0		1	1

Treatment outcomes of complete remission, partial remission, and stability were classified as effective, and treatment outcomes of progression were classified as ineffective. Effective was recorded as 1, and ineffective was recorded as 0.

### 2.3. Algorithm design

**Figure 1:**
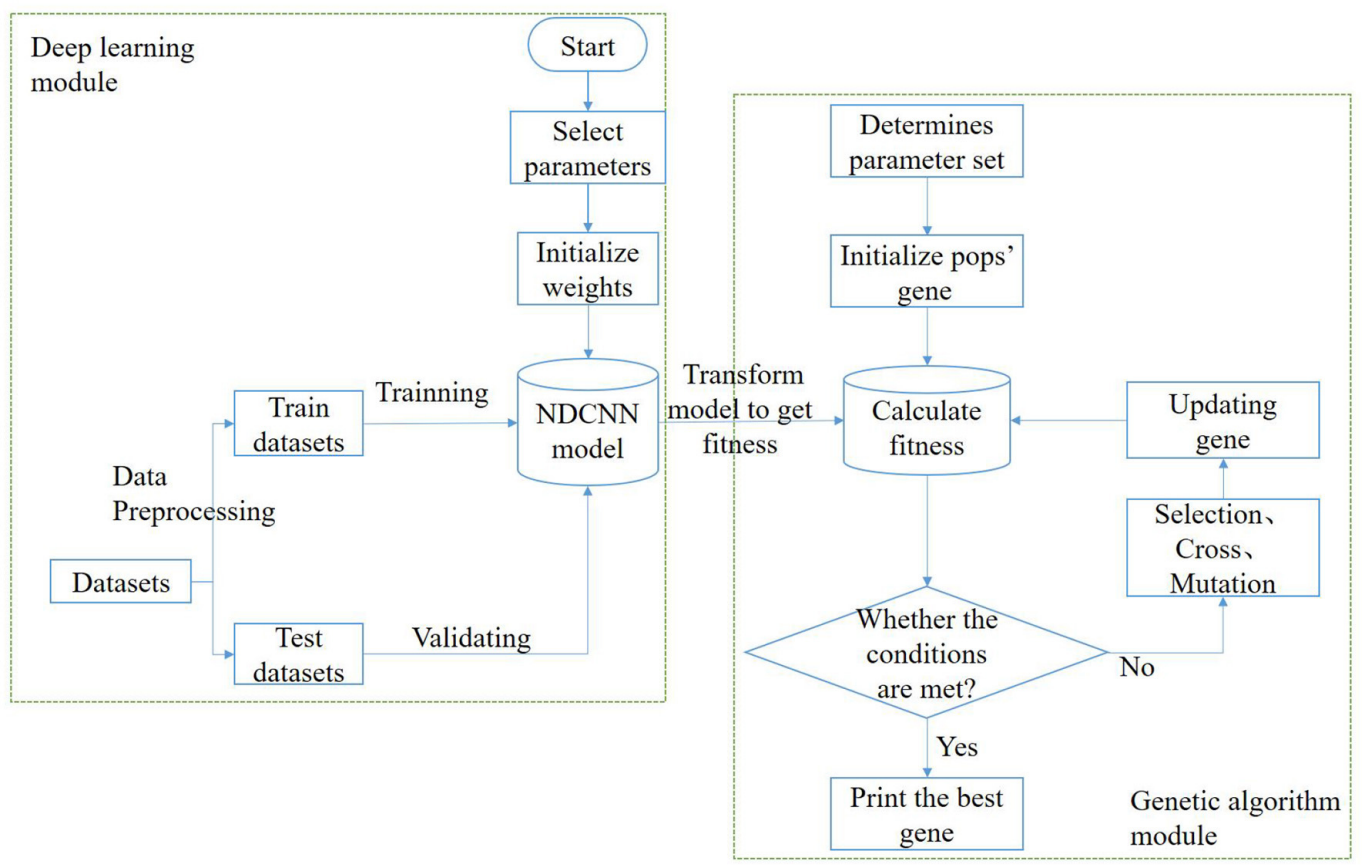
Algorithm flowchart.

In this task, the model needs to implement two functions: the first function is to predict the corresponding efficacy of any drug combination based on it, i.e., to determine whether a remedy can treat liver cancer; the second function is to generate multiple drug combinations based on certain design rules, i.e., to generate herbal combinations in a certain combination. Therefore, to achieve the above two types of functions, the algorithmic model used in this study should be divided into two parts: a deep learning module and a genetic algorithm module (Figure 1).

**Figure 2:**
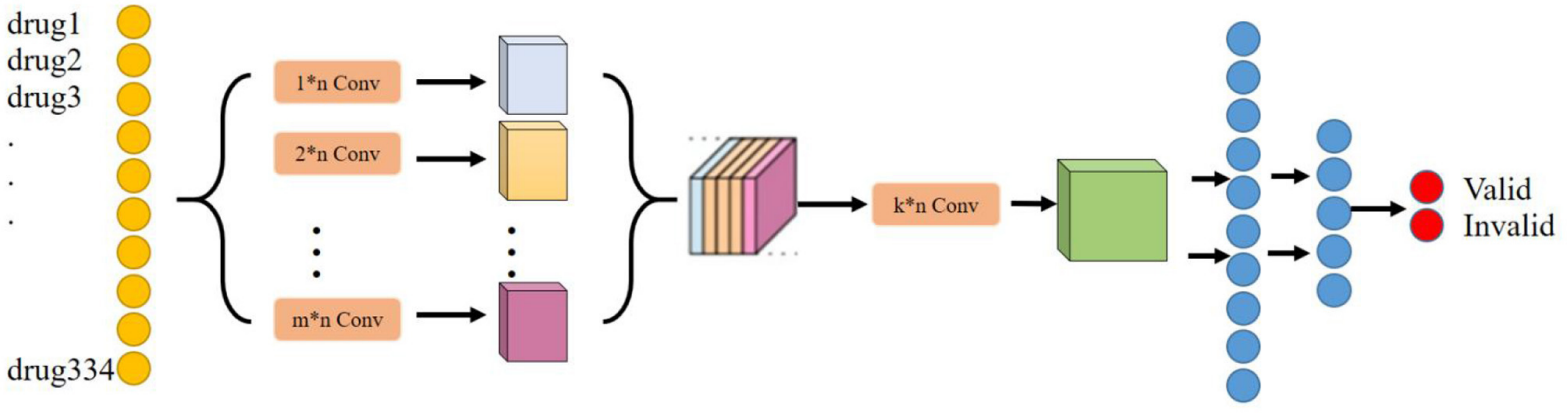
NDCNN model diagram.

In the deep learning module, the main purpose is to train and obtain a deep learning model that can predict the effectiveness of the treatment of liver cancer, i.e., to achieve the first function discussed above. The process roughly consists of the following steps: First, the dataset is preprocessed and vectorized into a training set and a test set with an 8:2 ratio; second, the structure of the deep learning model is designed, determining the network layers, initializing several other hyperparameters such as the number of neurons, and selecting network weights and biases. Afterward, the deep learning model is trained using the training set until it satisfies the iteration conditions, after which the training is stopped, and the changing trend of some indicators is visualized during the training. Finally, the generalization ability of the deep learning model is verified using the test set to test the prediction effect of the model in the untrained dataset. The deep learning model selected for this module is NDCNN, and the structure is shown in Figure 2.

The genetic algorithm module's main purpose is to use natural evolution principles to obtain remedies or combinations of herbs that are more likely to treat liver cancer. This aims to achieve the discussed second function and can be roughly divided into the following steps: First, determine the population size, selection operator, crossover operator, and variation operator for the genetic algorithm. Second, initialize the genetic population, where each individual represents a drug combination. Each individual's fitness value is calculated based on the trained deep learning model's prediction of its effectiveness. Subsequently, evaluate whether the stopping condition of the genetic algorithm is met. Typically, this involves a maximum number of iterations. If the condition is met, the iteration is stopped. Otherwise, perform selection, crossover, and mutation operations on the genes of the current population to generate new individuals. Finally, obtain the combination of Chinese herbal medicines that is most likely to cure liver cancer.

#### 2.3.1. Deep learning module

The main purpose of the deep learning module is to determine the effect of any herbal combination that can predict the treatment of liver cancer. Considering that the deep learning model can tap deeper potential features to learn the mapping relationship between drug combination and effect, the deep learning model is chosen as the baseline model in this research. In addition, considering that drugs have a very strong correlation or rejection in the local association, a convolutional neural network layer has been chosen to extract this local feature. Moreover, considering that convolutional kernels of different scales can extract local features of different dimensions, we have chosen to use convolutional kernels of multiple scales or dimensions to extract richer local features. Therefore, NDCNN was finally chosen to be used in this study, and its model structure is shown in Figure 2.

##### 2.3.1.1. Network structure

Based on the basic principles of TCM treatment and the logical combining of drug screening, and combined with the basic network structure of the convolutional neural network, in this study, we have designed a neural network model (NDCNN) for TCM screening recommendation by using the TCM treatment plan and the treatment effect prescription in the case as the input and output of the network structure, respectively, and its structure is shown in Figure 2. It consists of *m* multilayer convolutional layers and *k* fully connected layers. The pre-processed TCM treatment information is imported into the convolutional layers as input data, and then, the multi-core convolutional layers are used to realize feature extraction for TCM treatment information and drug screening. Thereafter, the output function is used to perform non-linear output to obtain non-linear expression capability to fit the drug screening process. Figure 2 clearly shows that the NDCNN consists of several convolutional neural network layers of different scales, each of which uses a different size of the convolutional kernel to extract local features of different scales. In this task, the signaling of NDCNN consists of the following main parts:

Step 1: Feeding the model with a bag-of-words vector of drug combinations;
Step 2: Using multiple models of convolutional kernels in parallel to extract local features at different scales, respectively;
Step 3: Feature stitching of the local features at multiple scales to form a two-dimensional tensor matrix;
Step 4: Use multiple convolution kernels of the same model to perform convolution operations on the extracted two-dimensional tensor matrix to further obtain higher-order local features of different sizes to enrich the feature dimensionality of the model;
Step 5: Use multiple fully connected layers to synthetically learn the local features extracted in the previous stage, and the last layer uses the softmax function to normalize the output results to finally obtain the drug's effect on liver cancer.

The specific hyperparameters of the NDCNN model used in this study are shown in Table 2.

**Table 2:** NDCNN hyperparameters.

**Network structure**	**Parameters**
Number of iterations	25
Input vector dimension	334
First-class convolution kernel size, number of kernels, and activation function	1, 128, and Relu
The second class of convolution kernel size, number of kernels, and activation function	3, 128, and Relu
Class III convolutional kernel size, number of kernels, and activation functions	5, 128, and Relu
Second layer convolution kernel size, number of kernels, and activation function	3, 128, and Relu
Number of neurons (fully connected layer 1) and activation function	1,024 and Relu
Number of neurons (fully connected layer 2) and activation function	512 and Relu
Number of neurons (output layer) and activation function	2 and Softmax
Loss function	Cross-entropy
Optimizer	Adam Optimizer

Compared with traditional deep learning models, convolutional neural networks reduce the complexity of the network through three methods: sensory fielding, weight sharing, and downsampling. It usually consists of several parts: the input layer, the convolutional layer, the pooling layer, the fully connected layer, and the output layer. A training process can be divided into three steps: forward propagation, loss calculation, and backward propagation.

##### 2.3.1.2. Forward propagation

The focused process of the forward propagation algorithm for a convolutional neural network includes forward propagation for the input-oriented layer, forward propagation for the convolutional layer, and forward propagation for the pooling layer, while the propagation-oriented algorithm for the fully connected layer and output layer mainly uses Relu and Softmax activation functions. The forward propagation algorithms for the different networks are treated as follows:

In this task, the input of the network can be defined considering a total of 334 herbal medicines, so here, *n* = 334. The convolutional neural network operation is performed at three scales, specifically with the scale set to 1,3,5. At each scale, convolutional kernels for convolutional operation at all three scales are set to 128 and converted to one-dimensional local feature vectors. Subsequently, the output values of the neurons in the convolutional layer are defined as the neuron values. These values are determined by the weights, bias units, and dimensionality of the convolutional kernels. The neuronal operations of the convolutional layer can be described by the following equations:


(1) $$\begin{array}{l} a=\sigma \left(\sum_{k=0}^{m}W_{k}x_{k}+b\right) \end{array}$$

(2) $$\begin{array}{l} Relu\left(x\right)=max\left(0,x\right) \end{array}$$

The non-linear activation function in Equation (1), i.e., the ReLU function, defines the non-linear output of the neurons in the convolutional layer after the linear transformation, which can effectively perform gradient descent and thus make the convolutional network have better fitting and generalization capabilities.

Here, the input neuron dimension is set to K. Then, the output neuron value *a*^*L*+1^of the L+1st fully connected layer is defined as follows:


(3) $$\begin{array}{l} a^{L+1}=\sigma \left(\sum_{k}W_{k}^{L}a_{k}^{L}+b^{L}\right) \end{array}$$

The correct probability value of the output Chinese medicine name is set to P, and the formula is defined as follows:


(4) $$\begin{array}{l} P=sigmoid\left(z_{k}^{L}\right) \end{array}$$

where the f function is then used to output the predicted probability values and normalize the final output by the formula defined as follows:


(5) $$\begin{array}{l} sigmoid\left(x\right)=\frac{1}{1+e^{-x}} \end{array}$$

##### 2.3.1.3. Loss function

In the deep learning module, the main objective is to obtain a model that can predict the effect of any combination of drugs in the treatment of liver cancer, which is a binary classification task, and the final output is “effective” or “ineffective.” Then, the model will use the cross-entropy function as the loss value calculation model, which can effectively avoid the problem that the gradient is too small when using the squared difference loss function, which prevents the network from further training. The cross-entropy loss function L is provided by the formula (6):


(6) $$\begin{array}{l} L=-\frac{1}{H}\sum_{h=1}^{H}\left(\left(t_{h}\log y_{h}+\left(1-t_{h}\right)\right)\log\left(1-y_{h}\right)\right) \end{array}$$

where H represents the number of neurons in the output layer, i.e., the number of herbal species to be screened. On the other hand, *t*_*h*_ ∈ {0, 1} represents the predicted value of the actual label, and *y*_*h*_(0 ≤ *y*_*h*_ ≤ 1) represents the predicted value of the model.

##### 2.3.1.4. Back propagation

The backpropagation algorithm in a convolutional neural network calculates the gradient of the loss function in the neural network with respect to each parameter in conjunction with an optimization method to update the parameters and reduce the loss function. Here, the error *δ* is used to represent the derivative of the loss function with respect to the current layer and the unactivated output *z*^*L*^, and *δ*^*L*^(*x*) represents the error *δ* at coordinate x in the Lth layer of the convolutional layer. Combining the chain derivative rule and the error *δ* in the Lth+1st layer, it can be assumed that the formula of *δ*^*L*^(*x*) is as follows:


(7) $$\begin{array}{l} \delta^{L}\left(x\right)=\sum_{dx}\delta^{L+1}\left(x-dx\right)W\left(x\right)\;\sigma^{{}^{\prime }}\left(z^{L}\left(x\right)\right) \end{array}$$

Then, based on the error *δ* in the Lth layer, the derivatives of that layer with respect to the weights and biases can be obtained as follows:


(8) $$\begin{array}{l} \frac{\partial C}{\partial w^{L}}=\delta^{l}*\;\sigma \left(z^{L-1}\right),\;\frac{\partial C}{\partial b^{L}}=\sum_{x}\delta^{L} \end{array}$$

Next, each of the networks in the parameter update method is set to the following equation:


(9) $$\begin{array}{l} W^{L}=W^{L}-\eta \sum \frac{\partial C}{\partial w^{L}} \end{array}$$

where η is the learning rate of the neural network, i.e., the magnitude of each parameter update. If the learning rate is set too large, the parameters to be optimized will fluctuate around the minimum value, and convergence will not be possible; if the learning rate is set too small, the parameters to be optimized will converge slowly, and the expected effect of the model will not be achieved as soon as possible. For the backpropagation of the fully connected layer, the following equation can be derived similarly:


(10) $$\begin{array}{l} \delta^{L}={\left(W^{L+1}\right)}^{T}\delta^{L+1}⊙\sigma^{{}^{\prime }}\left(z^{L}\right) \end{array}$$

The subsequent weight update formula for the fully connected layer is handled in the same way as the convolutional layer, i.e., the input data are predicted using the forward propagation algorithm and the network's own parameters *W*^*L*^ and *b*^*L*^. The loss between the actual output value *t*_*h*_ and the network-predicted output value *y*_*h*_ is then calculated using the predicted and true labels substituted into the loss function, and finally, the loss value is back-propagated back to the model so as to update the network parameters and continuously improve the performance of the network in predicting the correct Chinese herbal medicine.

#### 2.3.2. Genetic algorithm module

The main purpose of the genetic algorithm module is to generate a drug combination based on certain rules by selecting several herbs from 334 drugs that have a high probability of treating liver cancer. The specific process of the genetic algorithm to find the best drug combination is shown below:

Step 1: Set the population parameters. The population parameters of the genetic algorithm mainly include population size, maximum number of evolutions, selection operator, crossover rate, variation rate, and fitness function. In our study, the population size was set to 10 individuals, the maximum number of evolutions was 1,000, the roulette wheel method was used as the selection operator, and the crossover rate and variation rate were both 0.1. The fitness function was particularly important here, and the fitness function cannot be obtained directly in this task formula, which consists of two parts; the first part is calculated from the value of the predicted drug treatment effect by the deep learning model, and the other part is calculated from the number of drug species, and its specific formula is shown in Equation (11), where N denotes the number of best drug species in the combination.
  (11) $$\begin{array}{l} fitness=P\left(NULL|X\right)+{\left(sum\left(X\right)-N\;\right)}^{2} \end{array}$$
Step 2: Initialize the population and calculate the current fitness value. Here, the population consisted of 10 individuals, each representing a drug combination, and the adaptation value was calculated for each individual. The smaller the value, the better.
Step 3: Select the operation to find the best individual globally. Here, the roulette wheel method was used to select 5 drug combinations individual fathers from which to enter the next step of the crossover and variation operation.
Step 4: Crossover and mutation operations for population evolution. Here, the parent genes were crossed two by two randomly to swap some genes. Then, the resulting offspring genes were mutated at some points randomly, and 0 became 1 or 1 became 0, thus reproducing new offspring genes to enrich the diversity of the species.
Step 5: Calculate the fitness value and determine whether the stop iteration condition is satisfied. Then, calculate the fitness value of the new population of individuals and then judge whether the current iteration meets the stopping condition. If it does meet the stopping condition, then it is prudent to end the iteration and save the most effective combination of therapeutic drugs. Otherwise, continue the iteration until the end condition is met.
Step 6: Output is the global optimal solution, i.e., the most likely combination of drugs to treat liver cancer.

### 2.4. Results

In this section, the trends in the evaluation metrics of the NDCNN model and genetic algorithm during the training process are described in detail.

#### 2.4.1. NDCNN model training process

In the training process of the NDCNN model, 80% of the data set was selected as the training set by random division, and the remaining 20% was used as the test set. At the same time, to prevent the phenomenon of overfitting the NDCNN model, the maximum number of iterations was set to 25, and accuracy was selected as the evaluation index. In each iteration of the NDCNN model, the training set was first used to complete it. In each iteration, the NDCNN model first uses the training set to complete the forward propagation of the signal and the backward feedback of the error and then uses the test set to test the current generalization performance of the model. The change curves of the loss value and accuracy of the training and test sets are collected throughout the iteration.

##### 2.4.1.1. Variation curves for loss values

**Figure 3:**
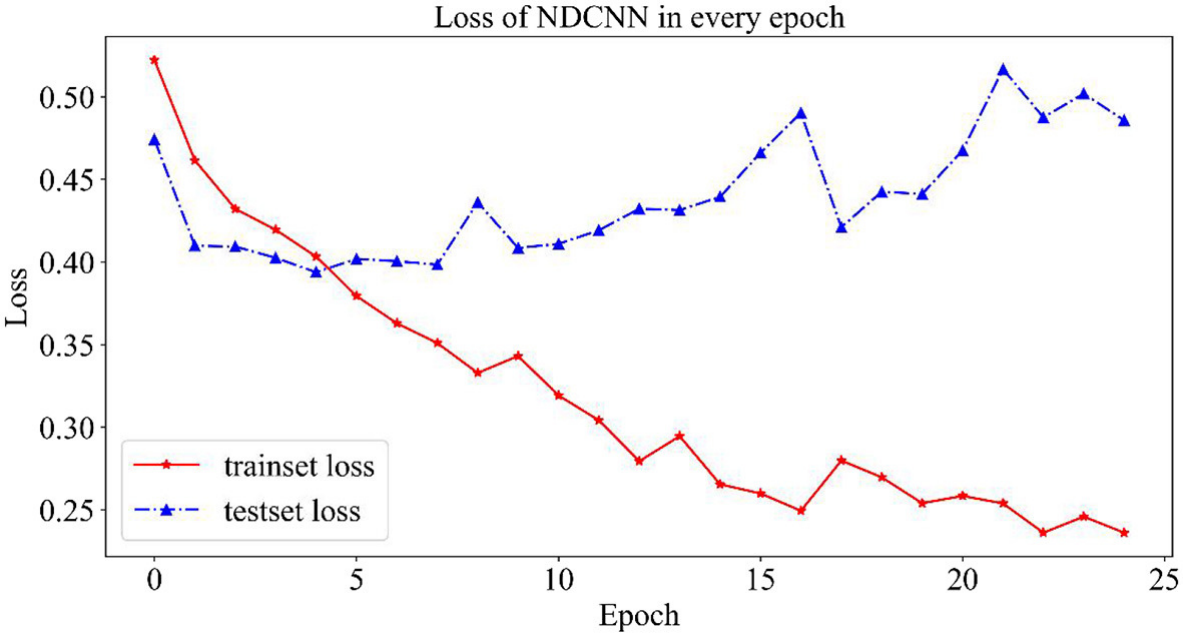
Loss value change curve of the NDCNN model.

As shown in Figure 3, the change curves for loss values on the NDCNN model for the training and test sets are demonstrated. From Figure 3, it can be observed that the loss value of the training set (red curve) gradually decreases during 25 iterations and does not tend to change smoothly; however, the loss value of the test set (blue curve) shows a trend of first decreasing and then increasing, and its value gradually decreases in the early stage as the model parameters keep iterating. The loss value of the test set is the smallest after five iterations, and its value starts to gradually become larger as the model parameters are further iterated. This indicates that, after five iterations, the model is in the best condition on the test set, and when the number of iterations is increased, the model starts to overfit. Therefore, the optimal number of iterations should be set to 5 when the model does not converge in the training set but achieves the best generalization ability in the test set.

##### 2.4.1.2. Change curve of accuracy rate

**Figure 4:**
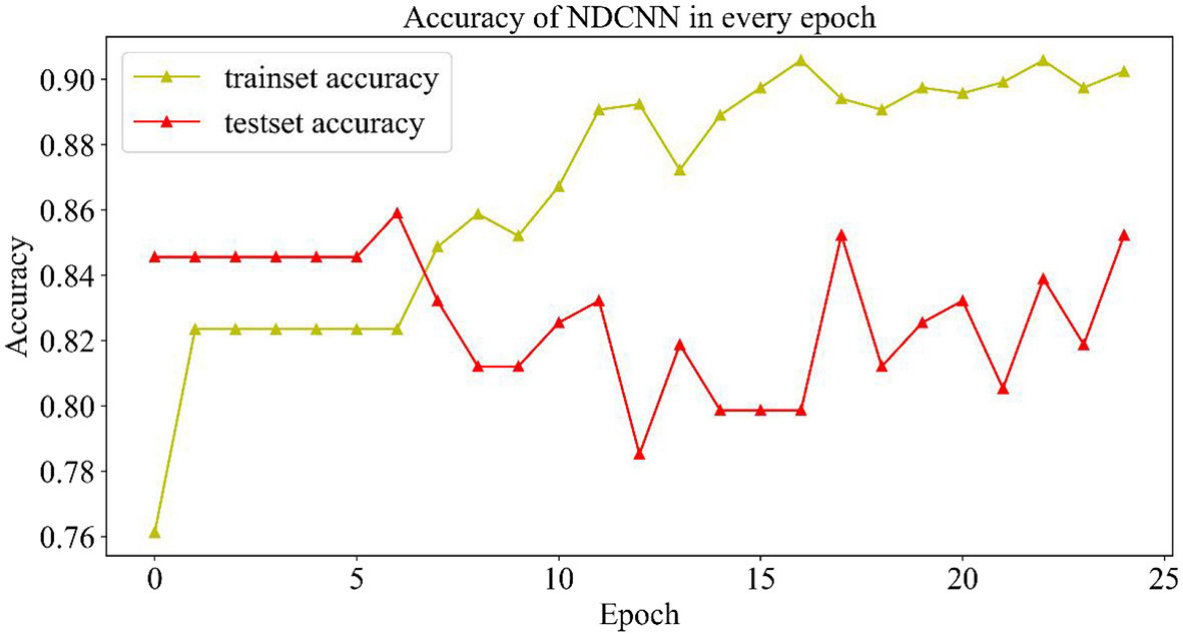
Accuracy variation curve of the NDCNN model.

As shown in Figure 4, the change curves of the training set and the test set during the iteration of the model are demonstrated. From Figure 4, it can be observed that the accuracy rate (yellow curve) of the training set gradually increases with the number of iterations and finally reaches ~0.9, indicating that the model gradually learns more potential features in the training set and can better achieve the mapping of features to labels, which can accurately predict the effect of ~90% of drugs. However, the accuracy change curve of the test set (the red curve) is exactly opposite to the change curve of its loss value. When the model iterates six times, it achieves the best generalization ability on the test set, but as the number of iterations increases, the model appears to overfit, and the accuracy of the test set starts to decrease and return to oscillation. Although the accuracy of the test set was repeatedly oscillating, it could still reach 0.84, indicating that the model could accurately predict ~84% of the new drug combinations, which indicates that the NDCNN model has strong generalization ability and can accomplish the task of predicting the effect of new drug combinations for liver cancer, which provides a prediction basis for the next step of using genetic algorithms to discover new drug combinations.

#### 2.4.2. Genetic algorithm optimization search process

The population size set here is 10, and the maximum number of iterations is 1,000, which means that, in each iteration, 10 individuals will be generated, corresponding to 10 drug combinations, and then, the NDCNN model is used to predict the efficacy of the 10 drug combinations. After obtaining the predictions, the fitness value of each combination is determined by considering the number of drug types combined with the prediction results. A smaller fitness value indicates a higher-quality individual, implying a greater likelihood of being selected and saved for the next iteration. The iterative process continues until either the maximum number of iterations is reached or a termination condition is met.

**Figure 5:**
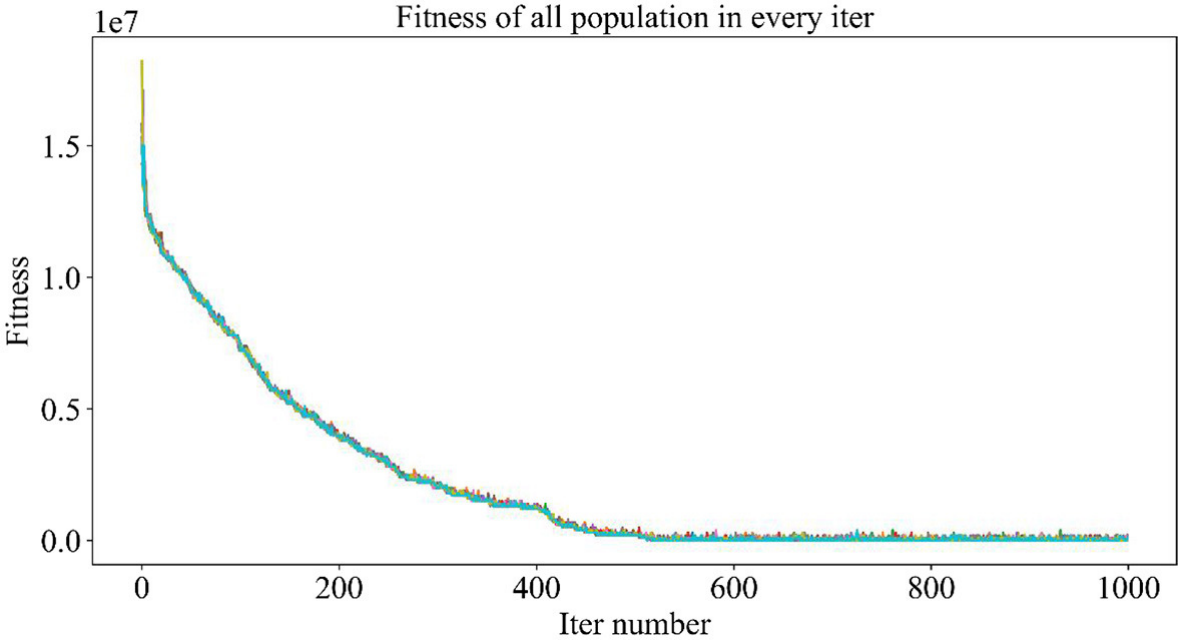
Adaptation trend of 10 individuals of the population during the iteration.

**Figure 6:**
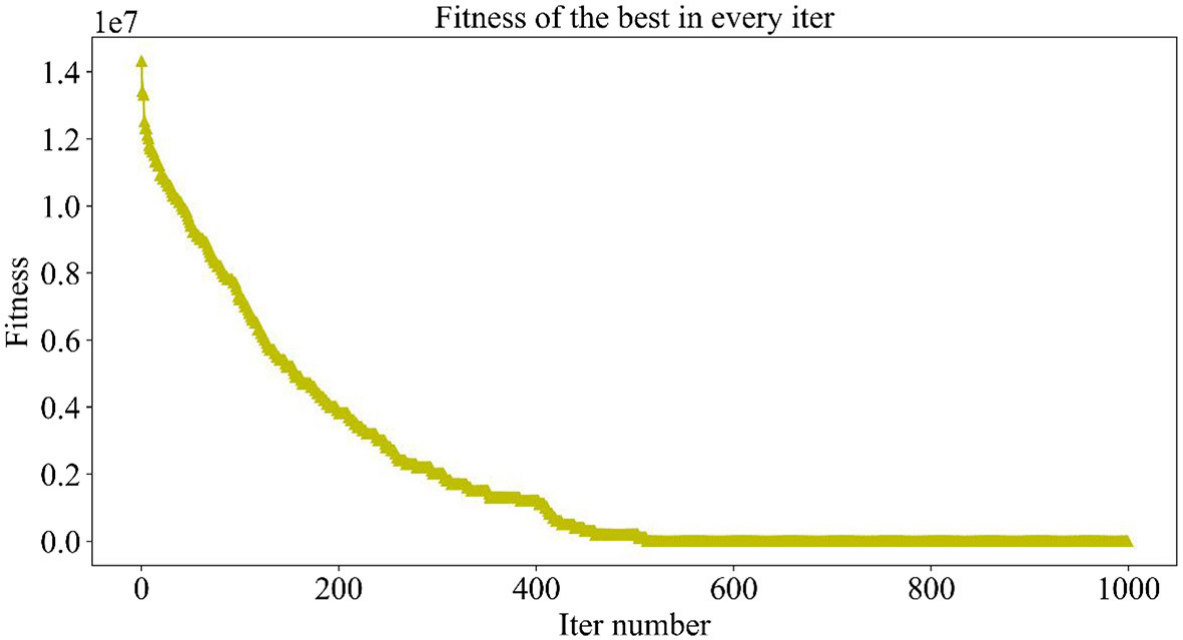
Trend of fitness of the global optimal solution in the population.

**Figure 7:**
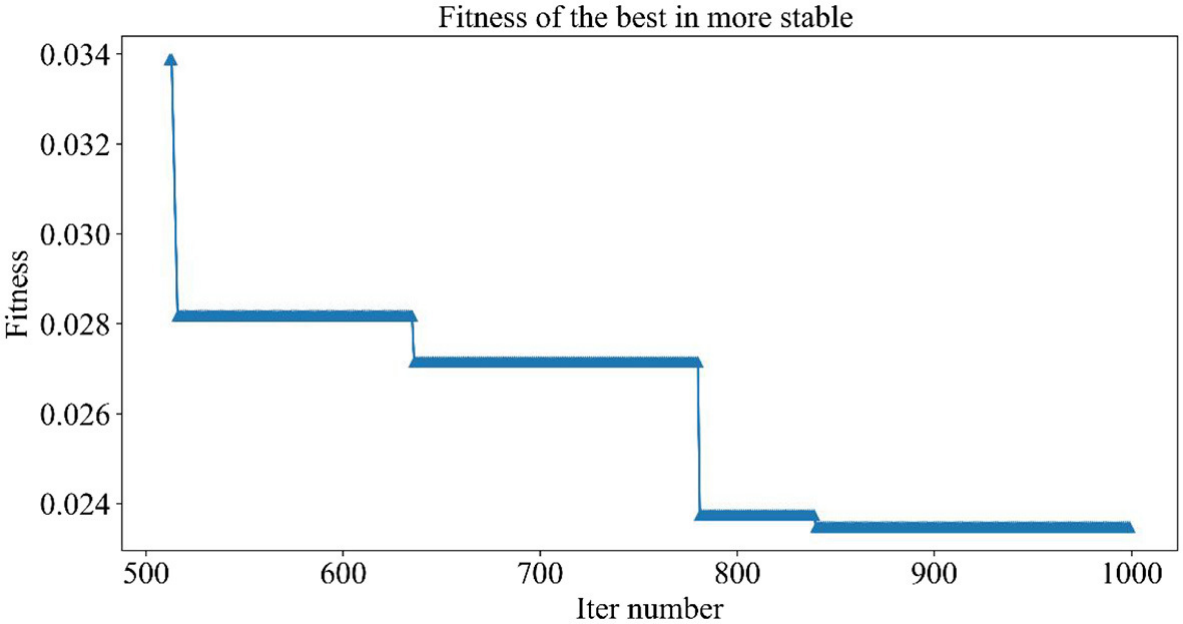
Trend of the global optimal solution for the population in the last 500 iterations.

Figure 5 demonstrates the trend of fitness values for all individuals in the population during each iteration. In Figure 6, the trend of fitness for the best individual in the population during each iteration is presented. Furthermore, Figure 7 depicts the trend of fitness for the best individual in the population, specifically over the last 500 iterations.

Figures 5, 6 show that the fitness values for all individuals in the population and the global optimal individuals gradually decreased and stabilized, which shows that the population individuals did not generate drug combinations according to the specified number of drug classes (here set to a maximum of 12 drug combinations) at the beginning. The number of drug classes of their individuals was much larger than the specified number, resulting in a very large value of fitness. To overcome this constraint, the genetic algorithm evolves gradually for approximately the first 500 iterations to mainly satisfy this condition. When the number of drug combinations remains at 12, the focus of the genetic algorithm shifts to finding the most effective drug combination, i.e., finding a certain combination of drugs that would make the NDCNN model predict “effective” with the highest probability. As can be observed from Figure 7, during approximately the last 500 iterations, the number of combined drug species is basically controlled within the set range, and the genetic algorithm starts to search for more effective drug combinations. After nearly 500 iterations, the global optimal individual was updated approximately “five” times, leading to the identification of the optimal drug combination.

#### 2.4.3. The most effective drug combinations searched for by the model

The best drug combination found using the genetic algorithm in this study was Strychnos nux- vomica L (Ma Qian Zi), Gleditsia sinensis Lam (Zao Jiao Ci), Psoralea corylifolia L (Bu Gu Zhi), Mylabris Phalerate Pallas (Ban Mao), Vaccaria segetalis (Wang Bu Liu Xing), Agrimonia pilosa (Xian He Cao), Juglans regia L (Fen Xin Mu), Paeonia lactiflora Pall (Bai Shao), Sargassum pallidum (Hai Zao), Paeonia suffruticosa Andr (Mu Dan Hua), Haliotis diversicolor Reeve (Shi Jue Ming), Roots of Paeonia suffruticosa Andr (Dan Pi). The probability that the NDCNN model considered this drug combination to be effective in treating liver cancer was 97.6%.

## 3. Bioconductivity analysis, network pharmacology analysis, and molecular docking

### 3.1. Data preparation

The RNA-seq high-throughput sequencing data and survival data of hepatocellular carcinoma patients in this study were obtained from the TCGA-LIHC dataset in the TCGA database (https://portal.gdc.cancer.gov), which contains 373 LIHC patients and 50 normal tissue controls. KM survival analysis was conducted separately for the 368 patients containing survival data and the 344 patients, including STAGE staging.

#### 3.1.1. PCA and difference analysis

PCA analysis was conducted after DESeq2 normalization of the downloaded expression profile counts data. In this study, we conducted a differential analysis using the R package DESeq2 to obtain differential genes between LIHC patients and normal controls, and genes with a fold change of >2, with a *P*-value of < 0.05, were identified as significantly differential genes and displayed using a volcano plot. The top 50 genes of difference were subsequently differentially displayed.

#### 3.1.2. Screening of active ingredients and target prediction of Chinese medicine compounds

Based on the conditions of oral utilization (OB) of ≥ 30% and drug-like properties (DL) of ≥ 0.18, the active ingredients and targets of the Chinese medicine compound were obtained from the TCMSP (https://tcmsp-e.com/browse.php?qc=ingredients) and herb databases (http://herb.ac.cn/). The drug targets were obtained by converting the UniProt database (https://www.uniprot.org/) to Gene Symbol and screening out duplicate values.

#### 3.1.3. Screening of common targets of Chinese medicine compounds for the treatment of hepatocellular carcinoma

The differential genes obtained in Step 2 intersected with the targets of the Chinese herbal medicine compound as the key genes for the efficacy of the Chinese herbal medicine compound in hepatocellular carcinoma. The Venn mapping tool (https://bioinfogp.cnb.csic.es/tools/venny/) was used to obtain the intersection set of Chinese herbal medicine components and disease targets.

#### 3.1.4. Construction of the active ingredient-target-disease network map

The software Cytoscape 3.8.2 was used to construct the network diagram. The active ingredient, the active ingredient's corresponding target, and liver cancer-related genes obtained using the above method were converted into working files, imported into Cytoscape software, and the corresponding parameters were adjusted appropriately to export the network target diagram.

#### 3.1.5. Construction of a PPI network map of common targets and network topology analysis

The intersecting targets were imported into the STRING database (https://cn.string-db.org/); the species “homo sapiens” was selected, and the confidence level of ≥ 0.9 was set as the screening condition; the key targets were sorted by degree, and the core targets were determined according to the ranking of the degree value. The higher ranking indicates that the protein involves a higher number of interactions and plays a more critical role in Chinese medicine. At the same time, the “Network Analyzer” tool was used to screen the active targets. The active targets were ranked according to the “degree” value from largest to smallest.

#### 3.1.6. GO enrichment analysis and KEGG enrichment analysis

GO and KEGG pathway enrichment analysis was conducted using the R package Clusterprofile, with a *P*-value of < 0.05 as the screening condition.

#### 3.1.7. Molecular docking validation

The top six core targets of the seven screens were molecularly docked with the top five active ingredients of the seven screened herbal compound degrees. The small molecule compounds in sdf format were downloaded from the PubChem database (https://pubchem.ncbi.nlm.nih.gov/), and then, ChemBio3D was used to convert the compounds into mol2 format. Afterward, AutoDock software was used to convert the mol2 format compounds into pdbqt format compounds. The crystal structure of the protein was downloaded from the PDB database (http://www.rcsb.org/) and then pre-treated with AutoDockTools software for dehydration, hydrogenation, and so on. Molecular docking was performed with the help of AutoDock Vina, and visualization was performed using Discovery Studio.

#### 3.1.8. Survival analysis and single-factor cox analysis

In this study, we used the R package SURVIVAL to analyze the relationship between survival time, survival status, gene expression profiles, and hepatocellular carcinoma. We utilized the Kaplan–Meier method to plot survival curves. Additionally, we assessed the impact of genes influenced by herbal compounds on the overall survival of patients using the one-factor Cox method.

## 4. Results

### 4.1. Analysis of variance

**Figure 8:**
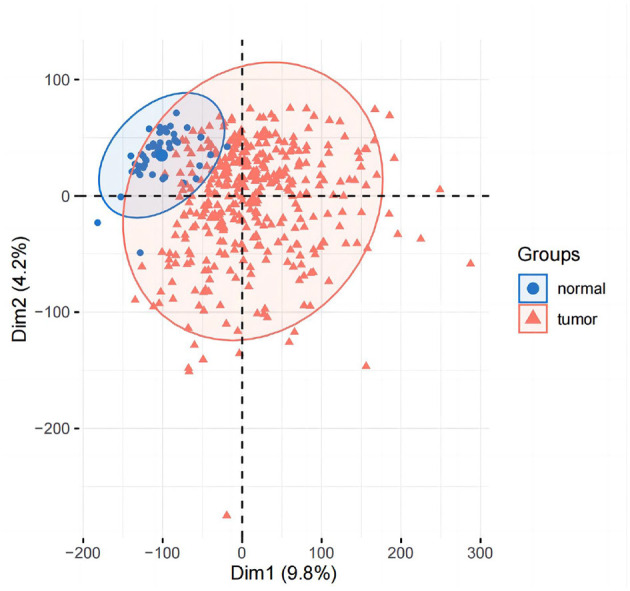
PCA analysis graph.

**Figure 9:**
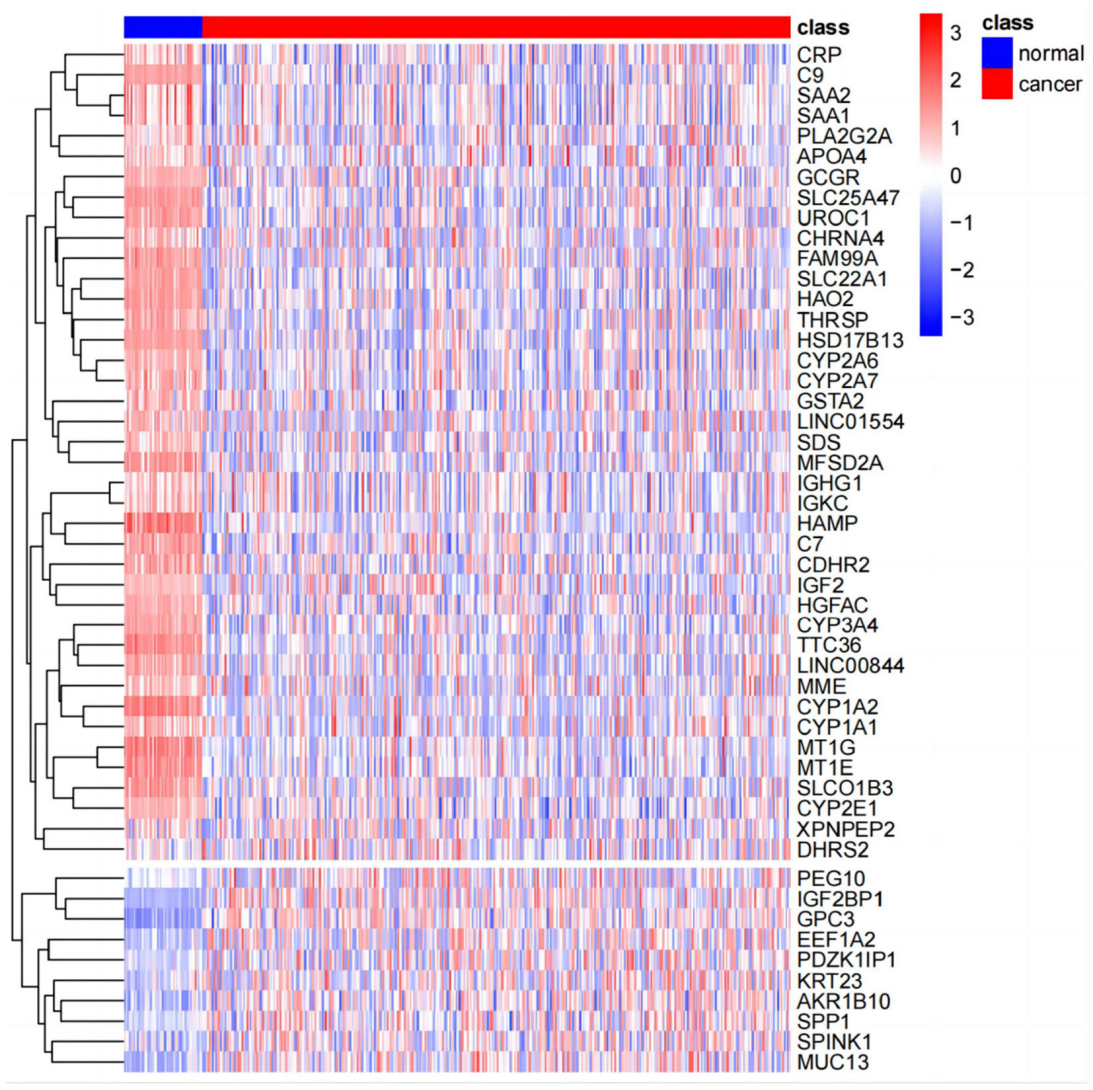
Gene heat map (TOP50).

The results of principal component analysis (PCA) are shown in Figure 8. In the analysis of variance, we found 1,936 upregulated genes and 7,053 downregulated genes in LIHC patients compared to normal controls (fold change >2, *P*-value < 0.05). The heat map of genes with significant differences TOP50 is shown in Figure 9.

### 4.2. Screening results of the active compounds

The TCMSP database was searched, and 14,71 chemical components of the Chinese medicine compound were obtained.

### 4.3. Prediction of potential targets for the treatment of hepatocellular carcinoma by TCMSP

**Figure 10:**
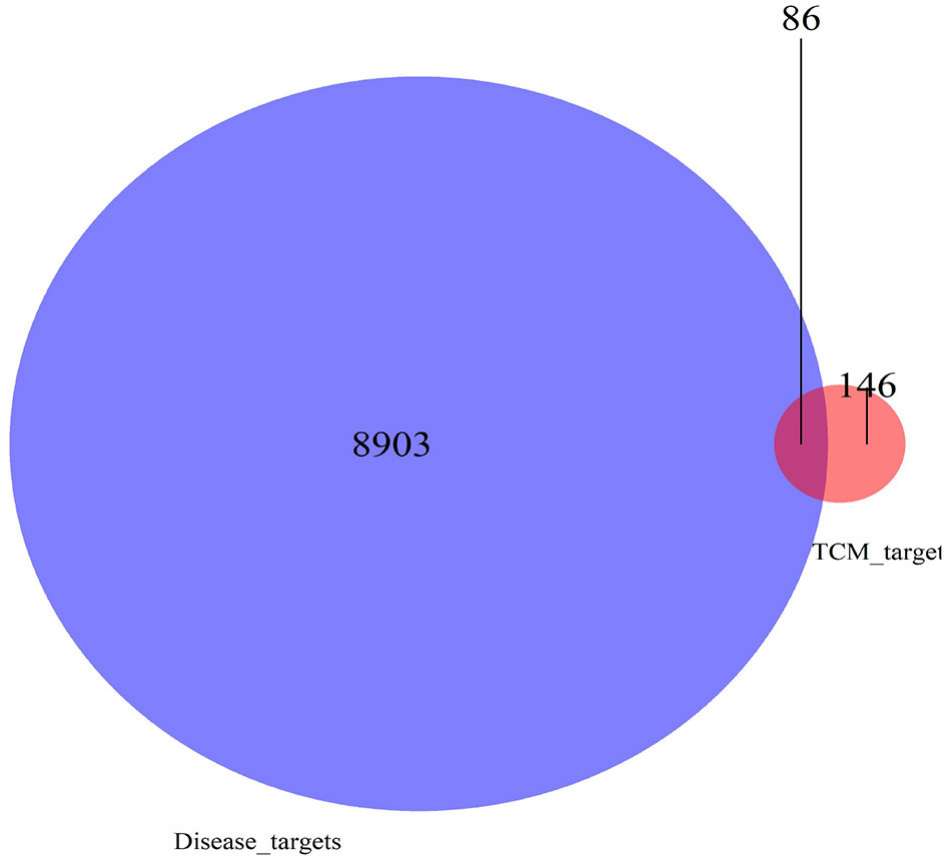
Venn diagram of active compound target-disease target intersection.

There were 39 active ingredients and 232 active ingredient targets in the TCMSP database. Based on the previous variance analysis, we concluded that there were 8,989 differential genes in liver cancer. By utilizing the Venny platform to take the intersection, it showed that there were 86 drug-disease intersection target genes between Chinese herbal compounds and liver cancer (see Figure 10).

### 4.4. Constructing a “disease-drug-active-ingredient-target” network

**Figure 11:**
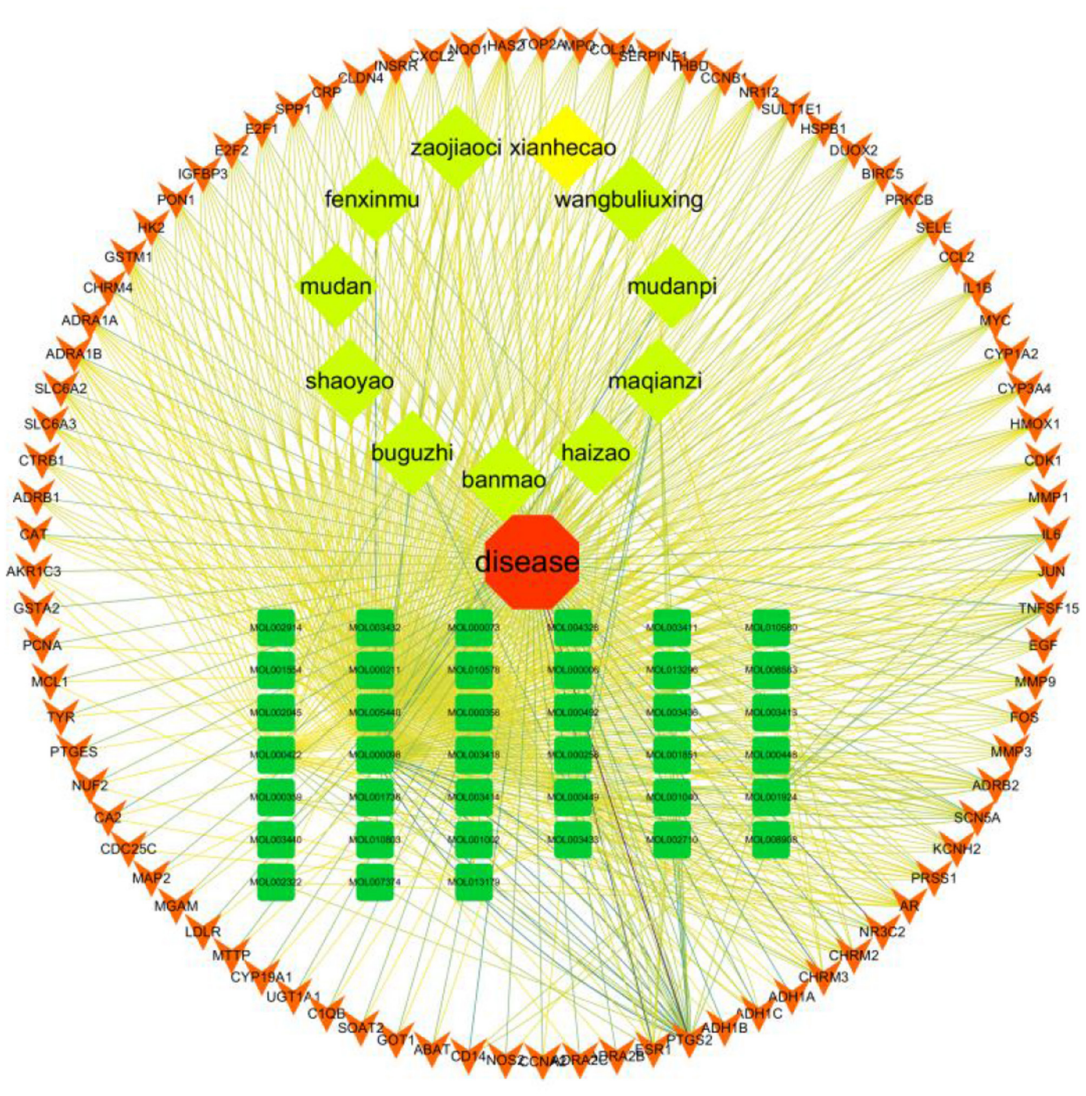
Compound herbal medicine - active ingredient - target - liver cancer network regulation map.

The “disease-drug-active-ingredient-target” network was constructed, as shown in Figure 11, and the topological properties of the network were analyzed using the Network analyze function, where the degree value is an important parameter to measure the criticality of a node in the network. The top 5 compounds in the network in terms of degree are quercetin, kaempferol, stigmasterol, luteolin, and (+)-catechin, which can connect to 831,162,109,53,27 targets, respectively, and are important for the treatment of liver cancer (Table 3).

**Table 3:** Basic information on key compounds.

**MOLID**	**Chemical composition**	**Degree**	**Source**
MOL000098	Quercetin	831	Hai Zao, Dan Pi, Wang Bu Liu Xing, Xian He Cao, Zao Jiao Ci, Fen Xin Mu
MOL000422	Kaempferol	162	Dan Pi, Xian He Cao, Zao Jiao Ci
MOL000449	Stigmasterol	109	Ma Qian Zi, Wang Bu Liu Xing, Zao Jiao Ci, Bu Gu Zhi
MOL000006	Luteolin	53	Xian He Cao
MOL000492	(+)-catechi	27	Ma Qian Zi, Dan Pi, Xian He Cao

### 4.5. Results of the PPI network and network topology analysis

**Figure 12:**
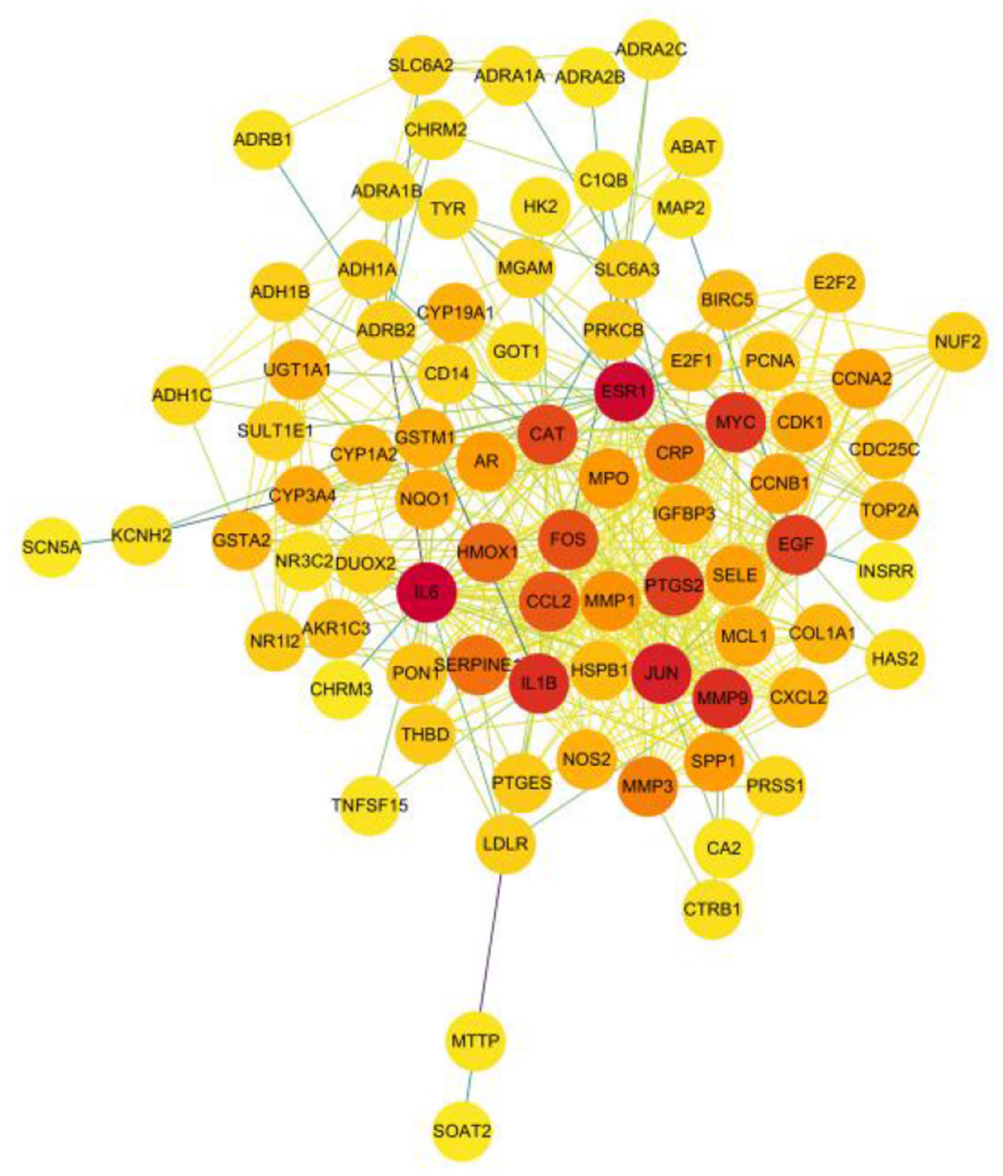
Protein interactions network of the herbal compound for liver cancer.

A total of 526 pairs of protein interactions were obtained from STRING database analysis with a minimum required interaction score of 0.9 as the screening parameter (Figure 12).

**Figure 13:**
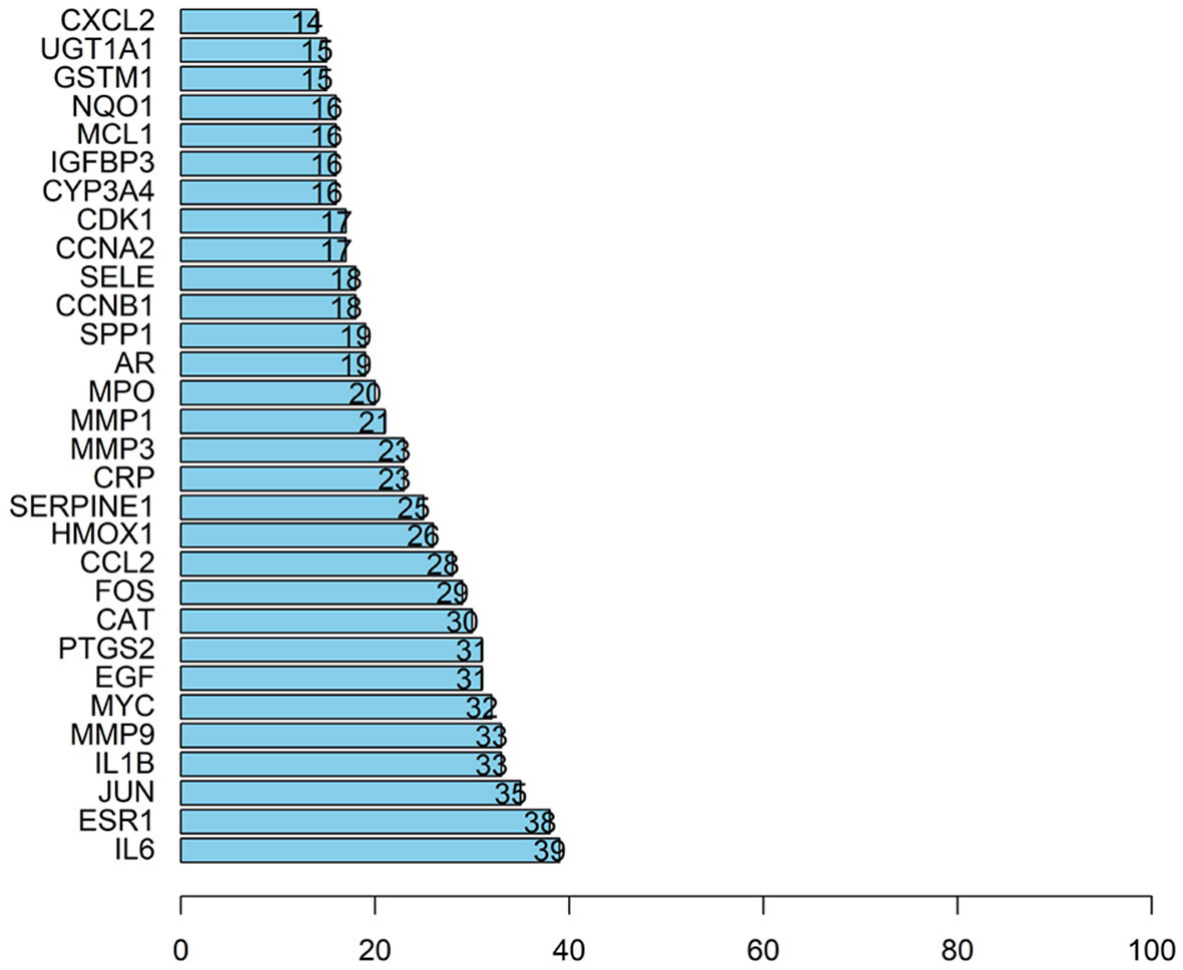
Ranking of node degree values of the protein interaction network of the herbal compound for liver cancer (degree value Top 30).

The article analyzed the degree values of the nodes in the network using the Network Analyzer tool and ranked the targets with degree values of ≥14 (mean value) from highest to lowest (see Figure 11). The top five protein genes with degree values were Interleukin-6 (IL-6), Estrogen Receptor 1 (ESR1), Proto-oncogene proteins (JUN), Interleukin-1 beta (IL-1β), and Matrix metalloproteinase-9 (MMP9). These proteins play a key role in the whole network, and the targets corresponding to the proteins play an important role in the treatment of hepatocellular carcinoma by herbal compounding, which is considered the key target of herbal compounding for hepatocellular carcinoma (Figure 13).

### 4.6. Results of the GO enrichment analysis

**Figure 14:**
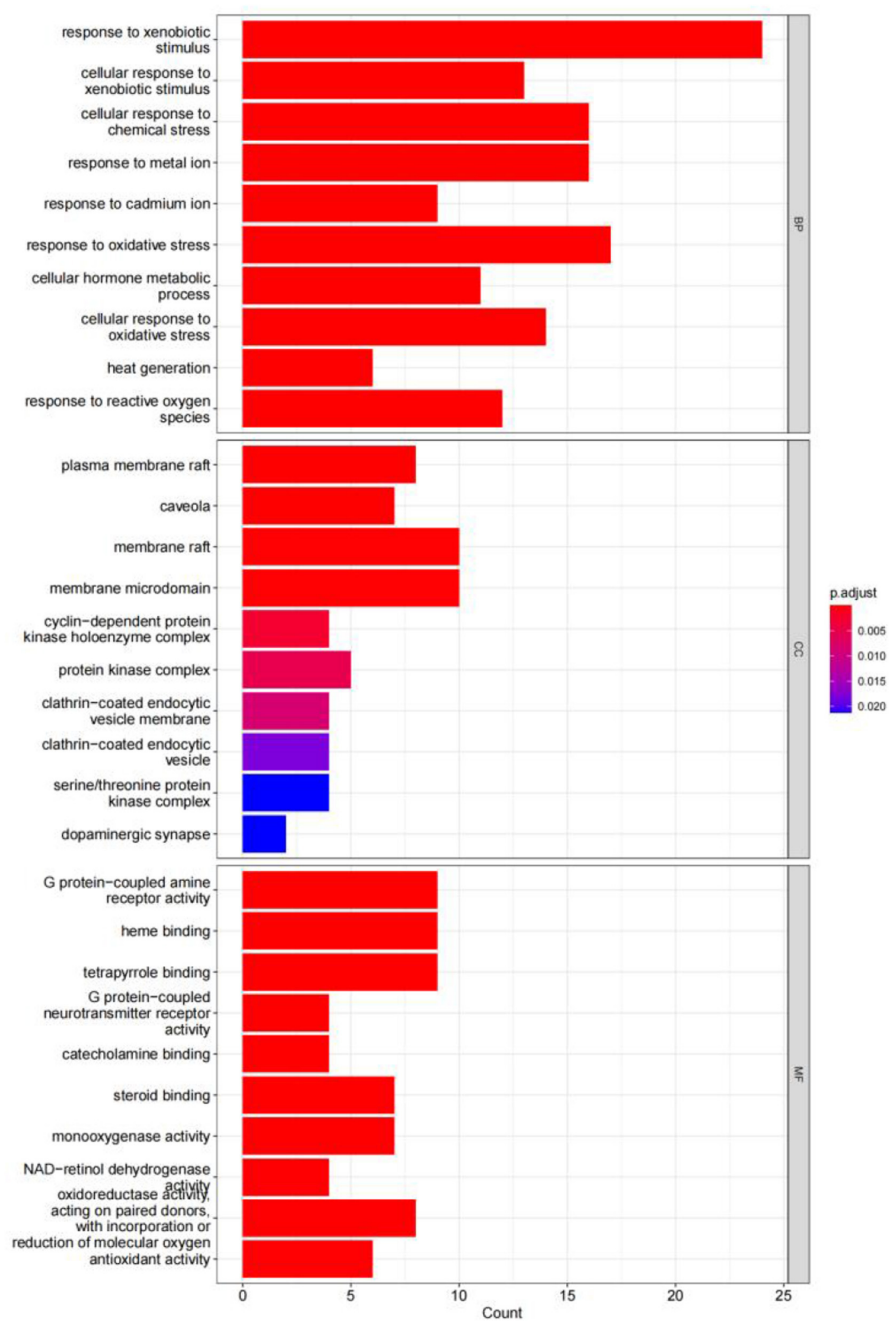
GO functional enrichment analysis (Top 10).

The GO enrichment analysis yielded a total of 820 GO entries (*P* < 0.01), including 748,12,60 entries for biological process (BP), cellular composition (CC), and molecular function (MF), respectively. From the analysis of the relevant entries of biological processes, the targets of herbal compound treatment for liver cancer were mainly involved in the cellular response to the xenobiotic stimulus, the cellular response to chemical stress, the response to the metal ion, the response to oxidative stress, and other processes; from the analysis of the relevant entries of cell composition (CC), the therapeutic targets mainly include the plasma membrane raft, the protein kinase complex, the membrane microdomain, and other regions. Through the analysis of molecular function entries, we identified the therapeutic targets primarily associated with G protein-coupled amine receptor activity, tetrapyrrole binding, and monooxygenase activity. The top 10 entries of each module, sorted by *P*-value, are shown in Figure 14.

### 4.7. Results of KEGG enrichment analysis

**Figure 15:**
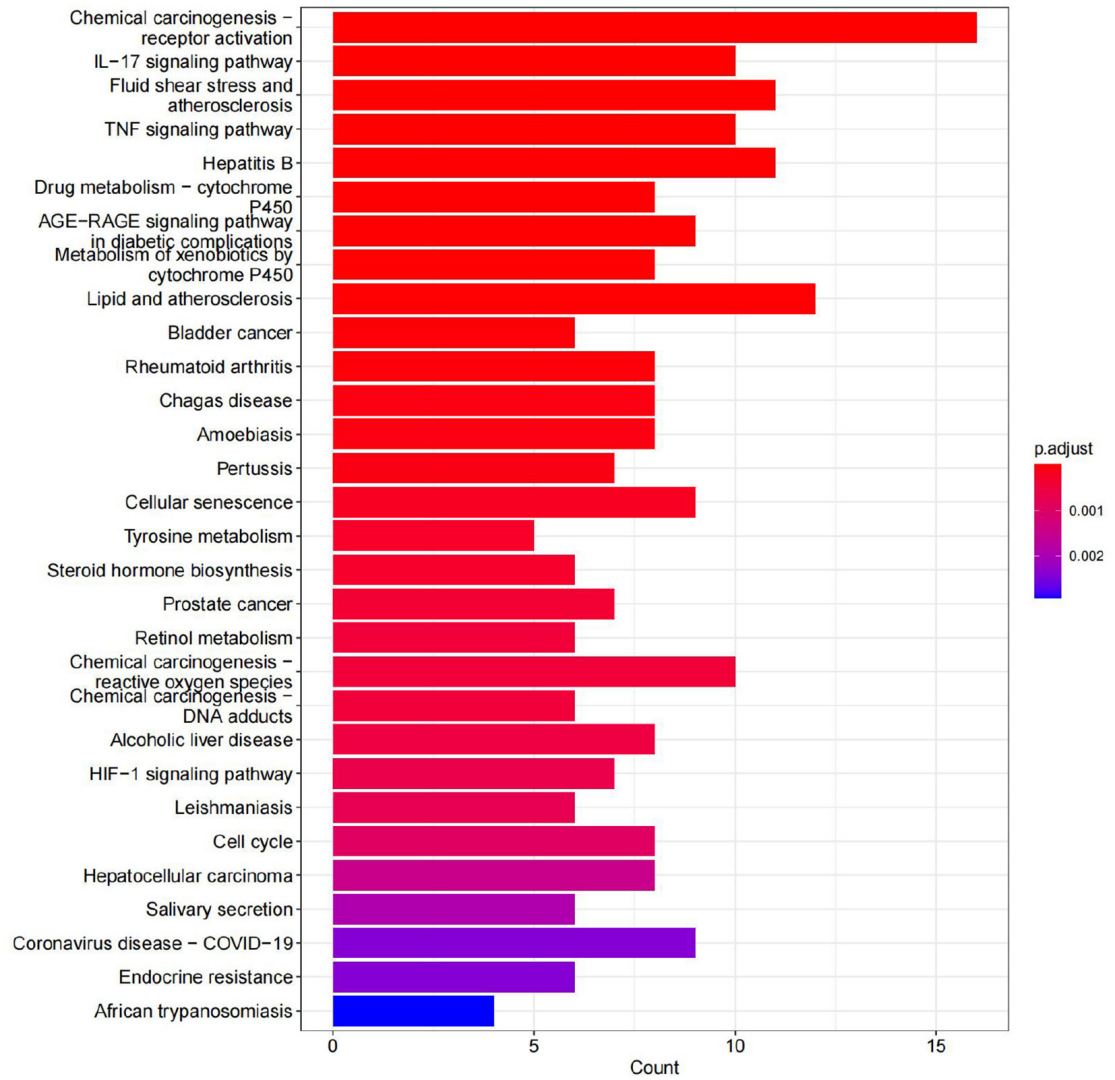
KEGG signaling pathway (Top 30).

A total of 70 pathways were obtained from the KEGG pathway enrichment analysis, and the top 20 entries were listed according to their *P*-values. Among these pathways, the prominent ones include the IL-17 signaling pathway, the TNF signaling pathway, and chemical carcinogenesis. These pathways are closely related to the inflammatory response, immune regulation, atherosclerosis, hepatitis B virus replication, and so on (see Figure 15).

### 4.8. Molecular docking validation

**Figure 16:**
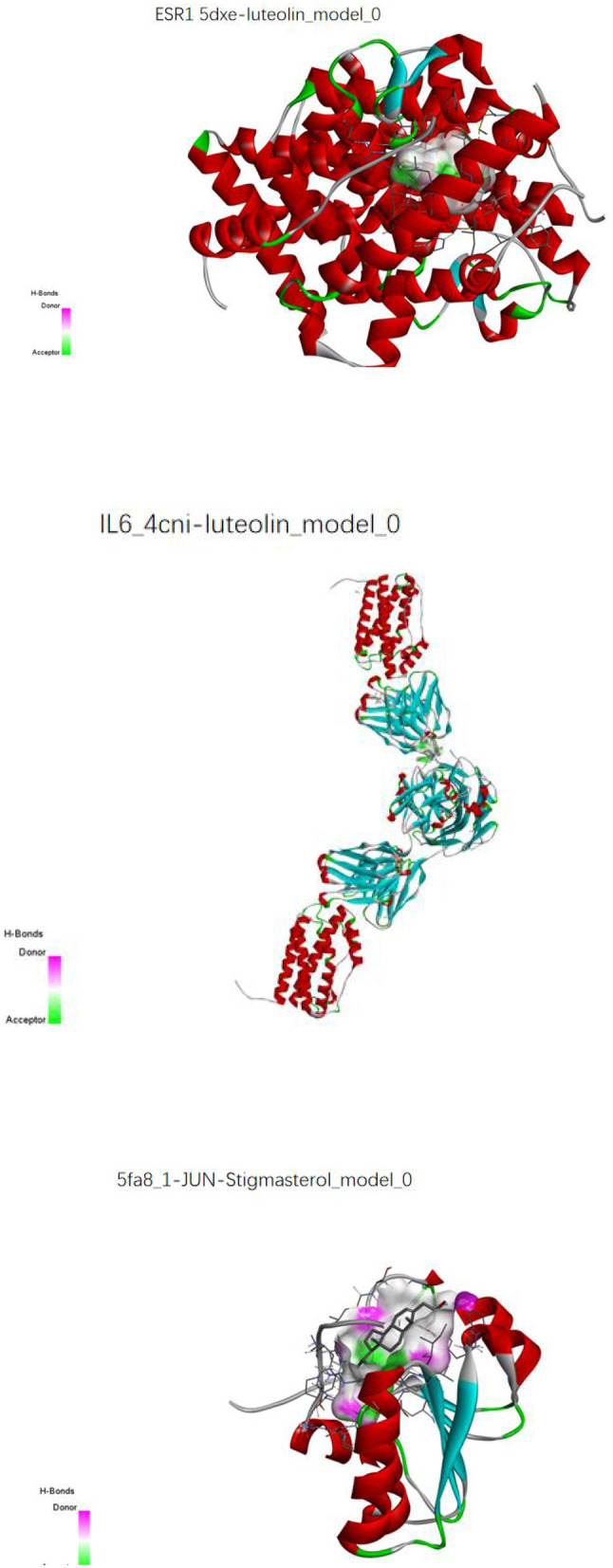
Molecular pair patterns of ESR1 and luteolin, IL6 and luteolin, and JUN and stigmasterol.

The key compounds screened in item “6.4” and the key target proteins in item “6.5” were verified by molecular docking. The molecular docking results showed that the molecular docking affinities of two key active compounds of the herbal compound, stigmasterol and luteolin, with the target proteins, were much <−5.0 k J/mol. As a result, ESR1 and luteolin, IL6 and luteolin, and JUN and stigmasterol showed good binding energy (Figure 16).

### 4.9. Survival analysis and one-way cox analysis

**Figure 17:**
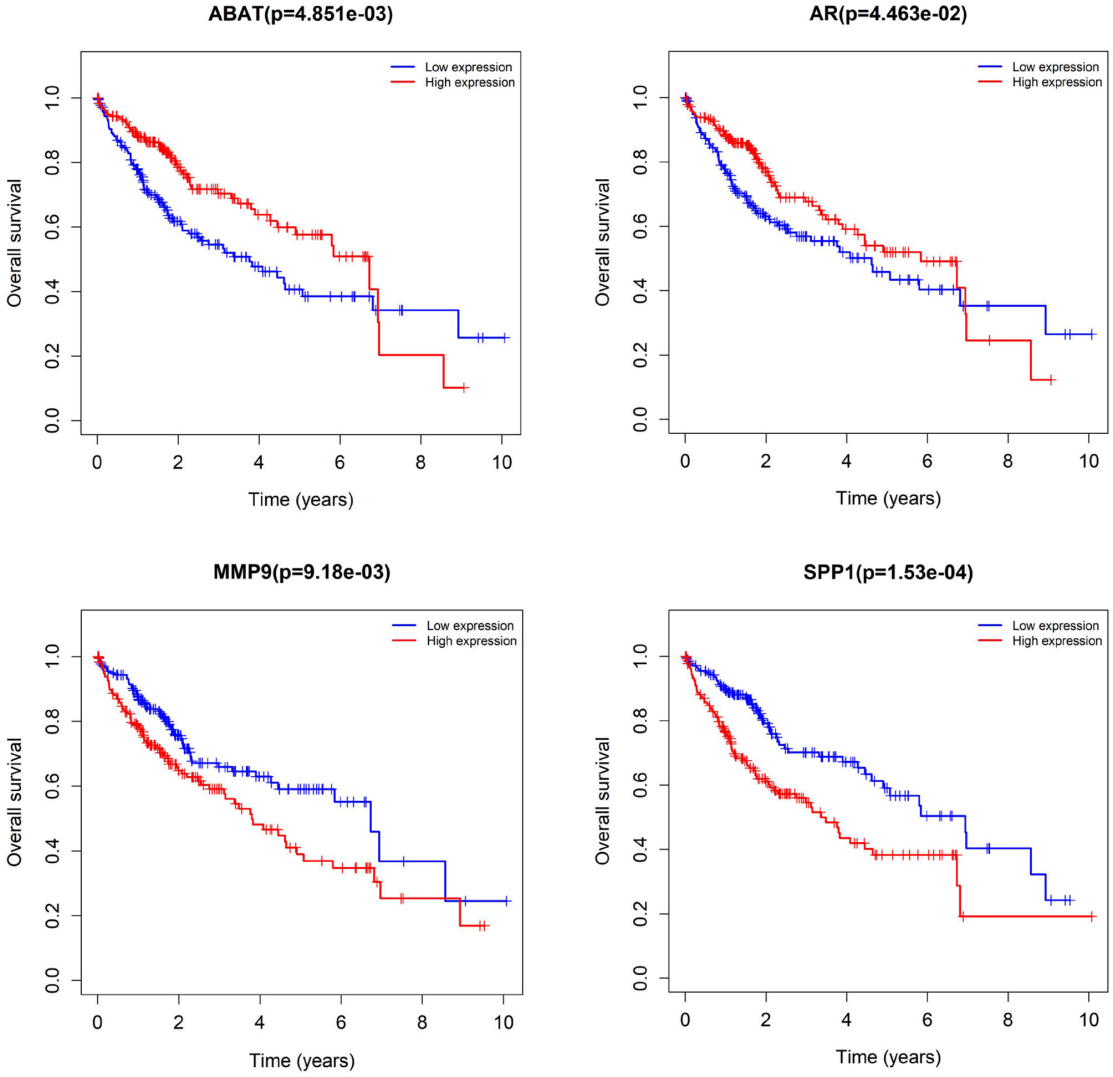
Examples of selected genes from survival analysis results.

**Figure 18:**
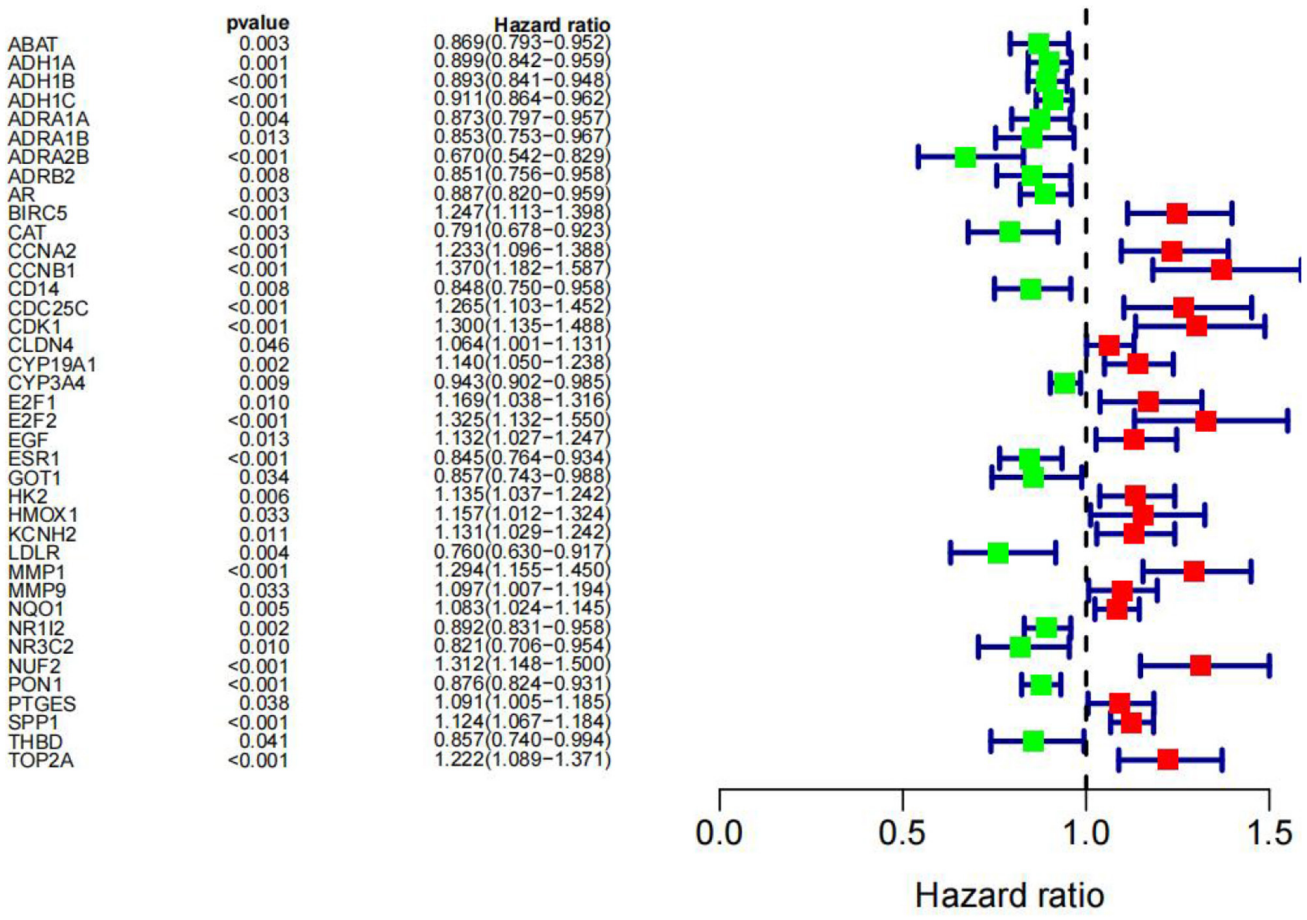
Graph of Cox analysis results.

We subjected 86 intersecting target genes of the herbal compound and hepatocellular carcinoma differential genes to survival analysis. The results suggested that 32 genes had a significant effect on prognosis (all *P* < 0.05) and found that AKR1C3, B1RC5, CCNA2, CCNB1, CDC25C, CDK1, CLDN4, CYP19A1, E2F1, E2F2, HK2, MGAM, MMP1, MMP9, NQO1, NUF2, SPP1, TOP2A, and 18 other genes with high expression were associated with poorer overall survival in hepatocellular carcinoma patients (log-rank *P* < 0.05), and ABAT, ADH1A, ADH1C, ADRA1A, ADRA1B, ADRA2B, AR, CAT, CD14, CYP3A4, ESR1, NR1L2, NR3C2, PON1, and 14 other genes with high expression were associated with longer overall survival in patients with hepatocellular carcinoma (log-rank *P* < 0.05). It showed that the above 32 genes were closely related to the prognosis of hepatocellular carcinoma patients (see Figures 17, 18).

## 5. Discussion

The attack on liver cancer has been a major challenge that needs to be urgently addressed by the World Health Organization. Surgical resection or liver transplantation is the most effective treatment for hepatocellular carcinoma (16). However, patients with hepatocellular carcinoma are often diagnosed at an intermediate to late stage, missing the best time for surgery, and the shortage of donors, high intraoperative risks, postoperative immune rejection, and expensive costs make the treatment of hepatocellular carcinoma limited. Although transhepatic artery chemoembolization, radiotherapy, chemotherapy, and targeted therapy for hepatocellular carcinoma are effective, the 5-year survival rate of hepatocellular carcinoma patients after treatment is still low (17). Therefore, there is an urgent need to find new treatment methods. Traditional Chinese medicine, a Chinese traditional medicine treatment method, has a long history of more than 2,000 years, and a large number of studies have shown that Chinese medicine has certain advantages in the treatment of tumor diseases (18, 19). The monomer or other active ingredients of Chinese medicine as natural drugs against tumors may form a trend in the future (20). For example, ginsenosides (21) and tanshinones (22) have been developed and applied in the treatment of colon and liver cancer, respectively. Clematis hederagenin saponin (CHS) in ginseng belongs to the triterpenoid saponins and can significantly induce apoptosis in breast cancer cells to exert anti-tumor effects (23). In this study, we screened an effective Chinese herbal compound for the clinical treatment of liver cancer using NDCNN combined with a genetic algorithm. Additionally, we conducted network pharmacology, molecular docking, and bioinformatics analyses to provide further evidence. The results demonstrated that the key chemical components of the screened Chinese herbal compounds could bind to key target proteins and may improve the prognosis of liver cancer patients by targeting several prognosis-related genes, thus aiming to contribute to the treatment of liver cancer.

Deep learning has the advantages of highly adaptive, denoising and data representation abilities. Cheng et al. (24) constructed the TextBLCNN model based on bidirectional long and short-term memory neural networks and convolutional neural networks to analyze the efficacy categories of 2,664 TCM prescriptions, and the final TextBLCNN model accuracy was 0.858. In this study, the NDCNN model was constructed based on TCM prescriptions and efficacy. The NDCNN model predicted whether the prescribed TCM could effectively treat liver cancer and improve the prognosis, and the accuracy of the predicted effect of the training set reached 90%, while the accuracy of the test set reached 0.84 after six iterations of the model test set. Although the phenomenon of overfitting appeared next, its accuracy could still reach 0.84, indicating that the NDCNN constructed in this study has good performance and certain clinical use values. The final combination of the genetic algorithm and the screened combination of Chinese herbal medicines [Strychnos nux- vomica L (Ma Qian Zi), Gleditsia sinensis Lam (Zao Jiao Ci), Psoralea corylifolia L (Bu Gu Zhi), Mylabris Phalerate Pallas (Ban Mao), Vaccaria segetalis (Wang Bu Liu Xing), Agrimonia pilosa (Xian He Cao), Juglans regia L (Fen Xin Mu), Paeonia lactiflora Pall (Bai Shao), Sargassum pallidum (Hai Zao), Paeonia suffruticosa Andr (Mu Dan Hua), Haliotis diversicolor Reeve (Shi Jue Ming), Roots of Paeonia suffruticosa Andr (Dan Pi)] implied that it could effectively treat liver cancer.

In our study, the difference analysis between liver cancer tissues and normal tissues revealed that liver cancer tissues had 8,989 differential genes compared to normal tissues, and we obtained 86 intersecting targets by taking the intersection with the targets of the herbal compound. Further studies revealed that quercetin, kaempferol, stigmasterol, luteolin, and (+)-catechin could match more disease targets. The core targets include IL-6, ESR1, JUN, IL1β, and MMP9, with complex and diverse inter-target action relationships. It is also enriched in multiple pathways, including the IL-17 signaling pathway, the TNF signaling pathway, chemical carcinogenesis receptor activation, Hepatitis B Cellular senescence, Fluid shear stress, and atherosclerosis signaling pathways. It is predicted that the herbal compound may treat hepatocellular carcinoma by regulating the inflammatory response, immune response, atherosclerosis, and so on. Therefore, it can be concluded that there is an associated synergistic effect among the active ingredients of herbal compounds, and their pharmacological targets play an important role in the treatment of hepatocellular carcinoma. The mechanism of hepatocellular carcinoma may be closely related to the inflammatory response, the immune response, and atherosclerosis. IL-17 is the signature cytokine of Th17 cells, and the IL-17 signaling pathway may be an important pathway regulating tumorigenesis progression. It promotes IL6 expression, and IL-6 promotes STAT3 phosphorylation, which activates the IL-6/STAT3 pathway and thus enhances the proliferation of hepatocellular carcinoma cells (25). TNF family genes play an important role in the regulation of cellular functions and in the proliferation and differentiation of immune cells; they can also act on the immune system in a co-inhibitory or co-stimulatory manner. The TNF family has been widely studied for its ability to enhance the immune response to tumors. This enhancement can be achieved through two mechanisms: increasing the signaling of members of the tumor necrosis factor receptor (TNFR) superfamily and using drugs to boost the immune response. By binding to TNF family members, these drugs have the potential to directly eliminate hepatocellular carcinoma cells and other types of tumors (26, 27). The KEGG pathway is also involved in the hepatitis B pathway, where hepatitis B virus (HBV) infection of the host results in the integration of HBV-DNA with host chromosomal DNA, leading to host gene rearrangement or even mutation, which destabilizes gene expression and leads to an imbalance in the interaction between proto-oncogenes and oncogenes and subsequently induces hepatocellular carcinoma (28). HBV-related proteins promote oxidative stress, leading to increased production of reactive oxygen species, which cause oxidative DNA damage, leading to increased genomic instability and subsequently inducing hepatocellular carcinoma formation (29). HBV-mediated immune response produces a large number of immune cells and inflammatory factors that constantly attack liver cells, leading to continuous liver inflammation and hepatocyte repair, resulting in liver fibrosis or cirrhosis, inducing liver cancer (30). HBV's X protein (HBx) performs a variety of biological functions, including transcriptional activation of various viral and cellular promoters, interaction with p53, interference with host DNA repair, and regulation of cell proliferation and apoptosis (31). One study (32) confirmed that HBx binds to calcium regulatory protein (CaM), promotes the release of Hsp90 from CaM, and activates LIMK1, which increases the phosphorylation of cofilin, a regulator of actin cytoskeletal reorganization, which in turn

promotes hepatocellular carcinoma metastasis, implying that the inhibition of the HBV replication pathway has the potential to improve the prognosis of hepatocellular carcinoma. As an important anti-tumor tool, cellular senescence can prevent the cellular replication of damaged DNA. Cellular senescence can be stimulated by a variety of mechanisms, such as telomere shortening, oncogene activation, DNA damage, intercellular fusion, and stresses that activate DNA damage response pathways (33). Together, these mechanisms limit excessive or abnormal cell proliferation, and, thus, the senescent state prevents cancer development. Accelerated cellular senescence is a new idea for potential strategies to treat liver cancer (34). A strong study confirmed (35) that accelerating tumor cell senescence can inhibit hepatocellular carcinoma cell proliferation, which in turn plays a role in improving the prognosis of hepatocellular carcinoma.

Quercetin is a flavonoid with antioxidant, anti-inflammatory, and immunomodulatory effects and can inhibit hepatocellular carcinoma growth and promote apoptosis by regulating inflammation, fibrosis, migration, apoptosis and angiogenesis, and oxidative stress and reducing components of the tumor microenvironment (36). Kaempferol is a flavonol compound mainly derived from the rhizome of the ginger plant Kaempferia. Stigmasterol has anti-inflammatory, antioxidant, anticancer, and other biological activities and pharmacological effects, and the combination of kaempferol and doxorubicin can inhibit the value-added migration and invasion of hepatocellular carcinoma cells (37). In addition, kaempferol can induce autophagy in hepatocellular carcinoma cells by regulating AMPK and AKT signaling molecules (38), and inhibiting the NF-κB pathway, which in turn inhibits hepatocellular carcinoma cell value addition and migration (39). Luteolin is a natural flavonoid with various pharmacological activities, such as anti-inflammatory, anti-allergic, and anti-tumor effects. It induces apoptosis and value-addition inhibition in hepatocellular carcinoma cells through the upregulation of oxidative stress and endoplasmic reticulum stress (40). By analyzing the key compounds, the article can suggest that there is a close synergistic effect among the active ingredients of the herbal compound, and the article achieves the effect of treating hepatocellular carcinoma by regulating biological processes such as inflammatory response, immune regulation, atherosclerosis, and hepatitis B virus replication. The molecular docking results showed that the binding energies obtained after the core target proteins were docked with the key active ingredients were all much less than the reference value of −5.0 k J/mol, indicating that the Chinese herbal compound could stably bind to the core target receptor protein of hepatocellular carcinoma and exert significant effects, and the network analysis results obtained based on this were of high credibility and reference value.

In conclusion, this study screened the herbal compound to improve the prognosis of liver cancer with the help of the NDCNN and a genetic algorithm and demonstrated the mechanisms through which the screened herbal compound treated liver cancer using network pharmacology, molecular docking, and bioinformatics. The herbal medicines screened using the NDCNN and the genetic algorithm in this study are not invariable; however, they will get different compositions of herbal medicines with each computer operation, but any herbal compound obtained may be better for the treatment of liver cancer. However, this study has some shortcomings and failed to further validate the network pharmacology-related signaling pathways through experiments. This is the focus of future research on the subject. More in-depth animal and cellular experiments will be conducted to verify the mechanism of action of the NDCNN-screened herbal compounds for the treatment of liver cancer to provide a more solid theoretical basis for the rational clinical application of herbal compounds.

## Data availability statement

The raw data supporting the conclusions of this article will be made available by the authors, without undue reservation.

## Ethics statement

Written informed consent was obtained from the individual(s) for the publication of any potentially identifiable images or data included in this article.

## Author contributions

ZC and XD designed the study. MW and RC performed the data collection and analysis. ZC wrote the manuscript. PP revised the manuscript. All authors contributed to the article and approved the submitted version.
